# Sebaceous carcinoma: clinical and demographic profile of patients in a tertiary referral hospital in Brazil^☆^

**DOI:** 10.1016/j.abd.2024.06.004

**Published:** 2025-01-17

**Authors:** Tatiana Mina Yendo, Flavia Mascarenhas Damiani, Suzana Matayoshi, Marcello Menta Simonsen Nico

**Affiliations:** a Department of Dermatology, Faculdade de Medicina, Universidade de São Paulo, São Paulo, SP, Brazil; b Department of Ophthalmology, Faculdade de Medicina, Universidade de São Paulo, São Paulo, SP, Brazil

*Dear Editor,*

Sebaceous carcinoma (SC) is a rare malignant neoplasm, described by Allaire in 1891, usually located in the head and neck and with a predilection for the periocular region (38.7%).^1^ It has a higher incidence in the elderly (6^th^ to 7^th^ decades of life), in males (58% of cases), and in Caucasian population (86.2%).^1^, ^2^ The authors retrospectively evaluated 22 cases of patients diagnosed with at least one SC, including three patients diagnosed with two primary SC, between January 2006 and December 2020, in a Brazilian university hospital. As described in previous studies,^2^ males had a higher incidence of SC, corresponding to 55% of the evaluated cases. The mean age at diagnosis was 67 years (24 to 94 years of age) and the majority of patients were Caucasian.

Clinically, the diagnosed SC showed varied characteristics, presenting as papules, plaques, nodules or tumors, generally yellowish or erythematous, with telangiectasias, a pearly gloss, keratosis or ulceration. The craniocephalic and cervical regions were preferentially affected, and, in ten cases, the SC was located in the periocular region (six in the upper eyelid and four in the lower eyelid; Fig. 1A). One case of SC was described in the neck, one in the ear pinna (Fig. 1B), three in the malar region, five in the nasal region, one in the scalp, one in the lip (Fig. 1C) and one in the chin. Only two SC were diagnosed outside the head and neck area (shoulder and chest). Nine patients were investigated for the presence of Muir-Torre Syndrome (MTS), and only four had the loss of expression of at least one mismatch repair gene confirmed by immunohistochemistry. Half of the studied cases had a diagnosis of other skin tumors such as basal cell carcinomas (BCC), squamous cell carcinomas (SCC), sebaceous adenomas and sebaceomas, single or multiple, with or without association with MTS. Moreover, eight cases had a diagnosis of non-skin neoplasms such as (1) invasive ductal breast carcinoma (without association with MTS), (2) Hodgkin's lymphoma, (3) non-Hodgkin's lymphoma and colon adenocarcinoma, (4) bilateral retinoblastoma (not investigated for MTS), (5) colon adenocarcinoma, (6) sigmoid neuroendocrine tumor and hepatocarcinoma, (7) colon adenocarcinoma, duodenal papilla adenocarcinoma with liver metastasis and prostate adenocarcinoma, (8) colon adenocarcinoma and multiple myeloma (confirmed for MTS).

**Fig. 1:**
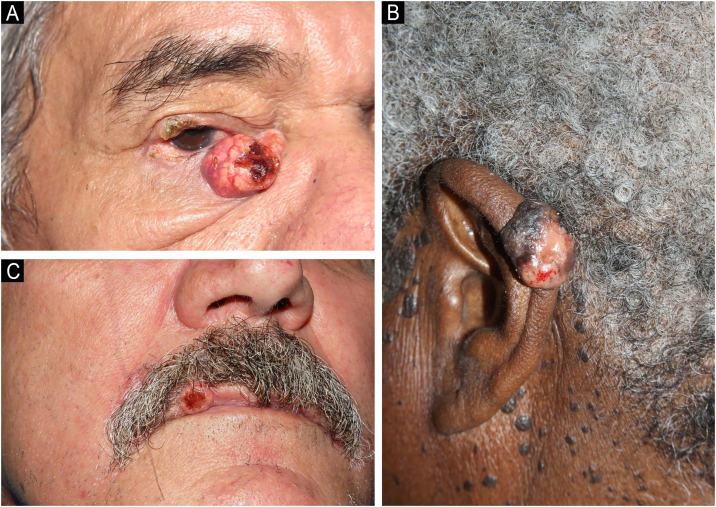
Clinical photographs of sebaceous carcinomas. (A) Sebaceous carcinoma in the lower eyelid. (B) Sebaceous carcinoma in the ear pinna. (C) Sebaceous carcinoma in the upper lip of a patient with MTS.

The treatment of choice in ten cases of SC was wide excision of the tumor. Mohs micrographic surgery was performed in one case, and in another case, radiotherapy was adopted as an adjuvant method. Only one case was treated with palliative radiotherapy. Four tumors were removed by shaving or saucerization. Patients mean follow-up time was four years, and only nine remained in outpatient follow-up until the end of the study. Three patients had SC metastasis: (1) one SC diagnosed in the lower eyelid, showed metastasis in a cervical lymph node, and wide excision was performed, without new recurrences; (2) two SC diagnosed in the upper and lower eyelids, with parotid and pulmonary metastases, were treated with excision of all tumors associated with radiotherapy, without recurrence; (3) one SC diagnosed in the shoulder, initially treated with wide excision, showed local recurrence one year later associated with lymph node metastasis, and died. There were also two deaths from causes unrelated to SC (hepatocarcinoma and multiple myeloma). The collected data are shown in Table 1.

**Table 1:** Clinical characteristics of patients diagnosed with sebaceous carcinoma.

Case N.	Gender	Age	Location	Tumor characteristics	Other skin tumors	Personal history	Muir-Torre Syndrome	Metastasis	Treatment	Recurrence	Follow-up (time in years)
1	M	51	Neck and lip	Erythematous papule and ulcerated yellowish papule	32 sebaceous adenomas and 1 sebaceoma	Colon adenocarcinoma	Confirmed: loss of MLH1 and PMS2 expression	Absent	Shaving	Absent	Outpatient follow-up (6)
2	F	94	Malar region	No description	2 BCC	NN	Absent	No description	Wide local excision	No description	Lost to follow-up (2)
3	M	64	Nose	No description	11 SCC, 7 BCC and 1 malignant sweat gland tumor	NN	Not investigated	Absent	No description	Absent	Outpatient follow-up (14)
4	M	79	Malar	Pearly keratotic papule	3 BCC	NN	Not investigated	No description	Wide local excision	No description	Lost to follow-up (<1)
5	M	60	Nose	Ulcerated erythematous plaque	1 BCC	Sigmoid neuroendocrine tumor and hepatocellular carcinoma	Confirmed: loss of MSH-2 and MSH-6 expression	Absent	Shaving	Absent	Death from hepatocarcinoma (2)
6	M	67	Scalp	Pearly erythematous tumor with telangiectasia	1 SCC	NN	Absent	No description	Wide local excision	No description	Lost to follow-up (<1)
7	F	61	Lower eyelid	Yellowish nodule with telangiectasias and ulceration	Absent	NN	Not investigated	Cervical lymph node	Wide local excision	Absent	Outpatient follow-up (5)
8	F	81	Upper eyelid	Reddish nodule with telangiectasias	Absent	NN	Not investigated	No description	Local excision	No description	Lost to follow-up (1)
9	M	65	Nose	No description	2 sebaceous adenomas and 1 SCC	Non-Hodgkin lymphoma and colon adenocarcinoma	Not investigated	No description	No description	No description	Lost to follow-up (<1)
10	F	69	Shoulder	Erythematous nodule with pearly gloss	1 BCC	NN	Not investigated	Lymph node	Wide local excision	Local recurrence after one year with lymph node involvement	Death (2)
11	F	86	Malar region	Keratotic plaque	2 SCC	NN	Not investigated	No description	Saucerization	No description	Lost to follow-up (<1)
12	F	64	Upper eyelid	No description	Absent	NN	Absent	Absent	Wide local excision	Absent	Outpatient follow-up (4)
13	M	71	Chest	Erythematous and grayish tumor	2 SCC	NN	Not investigated	Absent	Wide local excision	Absent	Outpatient follow-up (2)
14	M	67	Nose	Exophytic ulcerated nodule	Absent	Colon adenocarcinoma, duodenal papilla adenocarcinoma with liver metastasis and prostate adenocarcinoma	Confirmed: loss of MSH-2 and MSH-6 expression	Absent	Saucerization	Absent	Outpatient follow-up (<1)
15	F	24	Upper eyelid	No description	Absent	Bilateral retinoblastoma	Not investigated	No description	Local excision	No description	Lost to follow-up (<1)
16	M	52	Chin	Exophytic plaque	1 BCC, 1 sebaceoma and 2 sebaceous adenomas	Colon adenocarcinoma and multiple myeloma	Confirmed: loss of MSH-2 expression	No description	Wide local excision	Absent	Death from multiple myeloma (1)
17	F	74	Nose	Yellowish exophytic nodule with telangiectasias and ulceration	Absent	Hodgkin's lymphoma	Not investigated	No description	No description	No description	Lost to follow-up (<1)
18	F	82	Ear pinna	Keratotic nodule	Absent	Invasive ductal breast carcinoma	Absent	Absent	Mohs micrographic surgery	Absent	Outpatient follow-up (1)
19	M	62	Upper eyelid	No description	Absent	NN	Not investigated	No description	Unknown	Unknown	Lost to follow-up (<1)
20	M	71	Upper and lower eyelids	Yellowish ulcerated nodule	Absent	NN	Absent	Absent	Local excision and adjuvant RT	Absent	Outpatient follow-up (1)
21	F	43	Upper and lower eyelids	No description	Absent	NN	Not investigated	Parotid gland and lung	Excision of the primary tumor, parotid gland and lung metastases and RT	Absent	Outpatient follow-up (14)
22	M	93	Lower eyelid	Ulcerated plaque	Absent	NN	Not investigated	No description	Palliative RT	Unknown	Lost to follow-up (3)

BCC, Basal Cell Carcinoma; SCC, Squamous Cell Carcinoma; F, Female; M, Male; NN, Nothing Noteworthy; RT, Radiotherapy.

Morphologically, SC can present with varied characteristics, mimicking benign tumors (sebaceous hyperplasias, sebaceous adenomas, sebaceomas, nevocellular nevi), malignant neoplasms (BCC and SCC), as well as inflammatory diseases, such as chalazion, blepharitis and keratitis. The latter conditions are considered differential diagnoses of periocular lesions.^1^, ^3^ SC diagnosis is confirmed by histopathology of biopsies representing the deep dermis (Fig. 2) and immunohistochemical examination (positive EMA (epithelial membrane antigen) positive oil red O stain, positive Sudan black, with these last two stains performed on frozen sections; Fig. 3). Histopathology of SC is characterized by the presence of irregular and asymmetric sebaceous lobules; sebocytes present a vacuolated cytoplasm, hyperchromasia and nuclear atypia (Fig. 2).^2^, ^4^ Because it is rare and mimics other dermatoses, inadequate diagnostic procedures, such as shaving and saucerization, are performed, causing a further delay in the diagnosis of SC.

**Fig. 2:**
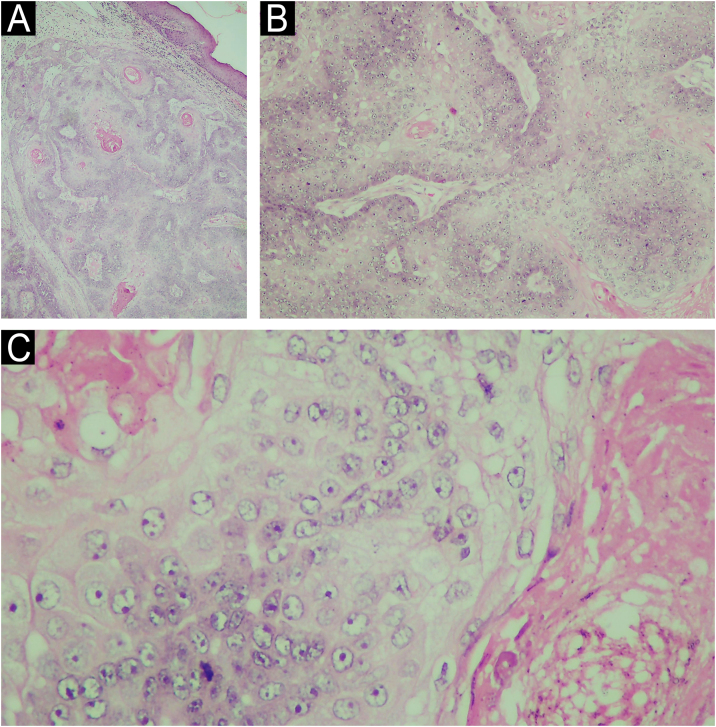
(A) Histopathology of sebaceous carcinoma in the left mandibular region (Hematoxylin & eosin, 10x). (B) Histological examination revealed the presence of disordered invasion of the dermis by ill-defined lobules of atypical sebocytes (Hematoxylin & eosin, ×100). (C) In a higher power view, atypical sebocytes can be observed in this tumor (Hematoxylin & eosin, ×400).

Most cases of SC occur sporadically and have as risk factors immunosuppression, especially solid organ transplantation and acquired immunodeficiency syndrome, exposure to ultraviolet (UV) radiation, radiotherapy, viral infections, a history of familial retinoblastoma and MTS.^5^ The predominance of SC lesions in photoexposed areas was documented in the present study, with 90% of the tumors located in the head and neck and 40% in the eyelid region (among them, two cases with lesions in the upper and lower eyelids). Another piece of evidence that the patients in the present sample had significant photodamage is the diagnosis of other UV-induced skin tumors, such as BCC and SCC, observed in 45% of the cases. Brazil is a country with a high incidence of UV radiation due to its geographic location, thus increasing the risk of the population for developing UV-induced skin malignant tumors.

MTS is a rare autosomal dominant disease in which there is a mutation in a DNA mismatch repair gene (MLH-1, MSH-2, MSH-6, PMS1 Homolog 2 and PMS-2),^4^, ^6^, ^7^ characterized by the presence of benign or malignant neoplasms originating in sebaceous glands that may or may not occur concomitantly with other malignant neoplasms, especially of the gastrointestinal tract and genitourinary system, the most common being colorectal cancer (80% of cases). Keratoacanthoma and BCC with sebaceous differentiation are also commonly diagnosed in these patients.^6^ In this disease, SC occurs at earlier age ranges and is common in extraocular regions.^8^ Suspicion of MTS based on the diagnosis of sebaceous tumors allows the early screening for visceral malignancies, which may favor a better prognosis for patients with this syndrome. In the present series, four cases were diagnosed with MTS, showing loss of expression of at least one DNA mismatch repair gene (Fig. 3) and with a history of colon neoplasia accompanied or not by another primary neoplasia. Only one case of MTS did not have another cutaneous neoplasia.

**Fig. 3:**
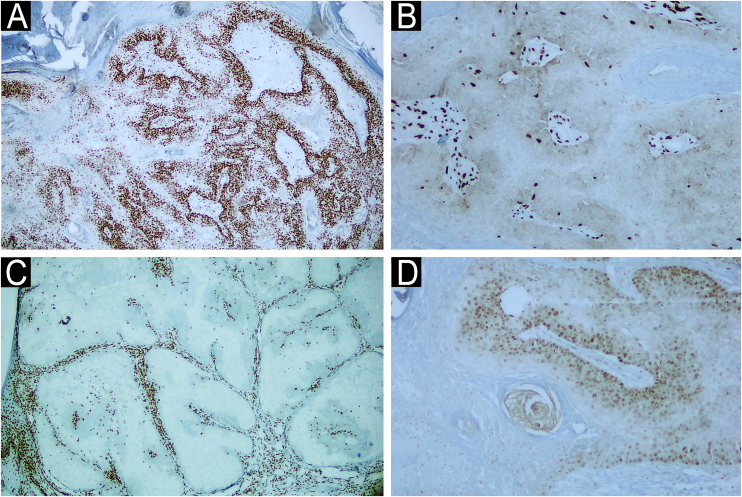
Immunohistochemical examination of a skin biopsy of sebaceous carcinoma from the left mandibular region of a patient with MTS. Immunohistochemical examination demonstrated the expression of mismatch repair genes (A) MLH-1 present (×40), (B) loss of MSH-2 (×400), (C) loss of MSH-6 (x40) and (D) loss of PMS-2 (x400).

Surgical treatment with tumor excision and intraoperative margin assessment is the most indicated for SC.^2^, ^9^ However, in periocular tumors, this can be challenging. In cases of margin involvement or locally advanced tumor with narrow margins, adjuvant radiotherapy may be indicated.^2^, ^10^ In the present series, there is a report of only one case treated with Mohs micrographic surgery (SC of the ear pinna) and one of adjuvant radiotherapy (SC of the upper and lower eyelids). Both patients were maintained on outpatient follow-up without recurrence of the primary tumor or metastases. The limited availability of trained professionals and equipment to perform Mohs micrographic surgery limits its use.

SC is an aggressive malignant tumor with high mortality (5%‒10%) and recurrence (16%‒18%) rates.^3^ Poor prognosis factors include tumors larger than 2 cm, multicentric disease, poorly differentiated lesions, and simultaneous involvement of the upper and lower eyelids.^9^ Three cases of metastases, one case of recurrence, and one death were observed in the present series, but these data were not sufficient to determine the metastasis, recurrence, and mortality rates, since among the limitations of the present study, the loss of patients to follow-up and incomplete documentation made it impossible to calculate them.

This retrospective case series discusses the importance of considering SC as a differential diagnosis, especially in periocular lesions. Early diagnosis, investigation of risk factors, and MTS are important to adopt the appropriate management for the benefit of the patient.

## Authors' contributions

Tatiana Mina Yendo: Design and planning of the study; collection, analysis and interpretation of data; drafting and editing of the manuscript, collection, analysis and interpretation of data; critical review of the literature; approval of the final version of the manuscript.

Flavia Mascarenhas Damiani: Collection, analysis and interpretation of data; statistical analysis; drafting and editing of the manuscript; collection, analysis and interpretation of data; critical review of the literature; approval of the final version of the manuscript.

Suzana Matayoshi: Critical review of important intellectual content; approval of the final version of the manuscript.

Marcello Menta Simonsen Nico: Design and planning of the study; critical review of important intellectual content; effective participation in research orientation; intellectual participation in the propaedeutic and/or therapeutic conduct of the studied cases; critical review of the literature; approval of the final version of the manuscript.

## Financial support

None declared.

## Conflicts of interest

None declared.
